# Investigation into the importance of using natural PVCs and pathological models for potential-based ECGI validation

**DOI:** 10.3389/fphys.2023.1198002

**Published:** 2023-05-18

**Authors:** Laura R. Bear, Jake A. Bergquist, Emma Abell, Hubert Cochet, Rob S. MacLeod, Remi Dubois, Yesim Serinagaoglu

**Affiliations:** ^1^ IHU-Liryc, Heart Rhythm Disease Institute, Foundation Bordeaux Université, Bordeaux, France; ^2^ University Bordeaux, CRCTB, Bordeaux, France; ^3^ INSERM, Centre de Recherche Cardio-Thoracique de Bordeaux, Bordeaux, France; ^4^ Scientific Computing and Imaging Institute, University of Utah, Salt LakeCity, UT, United States; ^5^ Norra Eccles Harrison Cardiovascular Research and Training Institute (CVRTI), University of Utah, Salt LakeCity, UT, United States; ^6^ Department of Biomedical Engineering, University of Utah, Salt LakeCity, UT, United States; ^7^ Bordeaux University Hospital (CHU), Pessac, France; ^8^ Electrical-Electronics Engineering Department, Middle East Technical University, Ankara, Türkiye

**Keywords:** ECGI, PVCs, hyperkalemia, inverse problem of electrocardiography, ECGI inverse problem, regularization

## Abstract

**Introduction:** Premature ventricular contractions (PVCs) are one of the most commonly targeted pathologies for ECGI validation, often through ventricular stimulation to mimic the ectopic beat. However, it remains unclear if such stimulated beats faithfully reproduce spontaneously occurring PVCs, particularly in the case of the R-on-T phenomenon. The objective of this study was to determine the differences in ECGI accuracy when reconstructing spontaneous PVCs as compared to ventricular-stimulated beats and to explore the impact of pathophysiological perturbation on this reconstruction accuracy.

**Methods:** Langendorff-perfused pig hearts (*n* = 3) were suspended in a human torso-shaped tank, and local hyperkalemia was induced through perfusion of a high-K^+^ solution (8 mM) into the LAD. Recordings were taken simultaneously from the heart and tank surfaces during ventricular pacing and during spontaneous PVCs (including R-on-T), both at baseline and high K^+^. Epicardial potentials were reconstructed from torso potentials using ECGI.

**Results:** Spontaneously occurring PVCs were better reconstructed than stimulated beats at baseline in terms of electrogram morphology [correlation coefficient (CC) = 0.74 ± 0.05 vs. CC = 0.60 ± 0.10], potential maps (CC = 0.61 ± 0.06 vs. CC = 0.51 ± 0.12), and activation time maps (CC = 0.86 ± 0.07 vs. 0.76 ± 0.10), though there was no difference in the localization error (LE) of epicardial origin (LE = 14 ± 6 vs. 15 ± 11 mm). High K^+^ perfusion reduced the accuracy of ECGI reconstructions in terms of electrogram morphology (CC = 0.68 ± 0.10) and AT maps (CC = 0.70 ± 0.12 and 0.59 ± 0.23) for isolated PVCs and paced beats, respectively. LE trended worse, but the change was not significant (LE = 17 ± 9 and 20 ± 12 mm). Spontaneous PVCs were less well when the R-on-T phenomenon occurred and the activation wavefronts encountered a line of block.

**Conclusion:** This study demonstrates the differences in ECGI accuracy between spontaneous PVCs and ventricular-paced beats. We also observed a reduction in this accuracy near regions of electrically inactive tissue. These results highlight the need for more physiologically realistic experimental models when evaluating the accuracy of ECGI methods. In particular, reconstruction accuracy needs to be further evaluated in the presence of R-on-T or isolated PVCs, particularly when encountering obstacles (functional or anatomical) which cause line of block and re-entry.

## 1 Introduction

Premature ventricular contractions (PVCs), caused by early activation of the ventricles, can lead to a range of complications, including PVC-mediated cardiomyopathy (Cha et al., 2012), ventricular tachycardia (John and Stevenson, 2016), and increased mortality risk in the presence of structural heart disease (Messineo, 1989). Catheter ablation of the site of origin can suppress isolated and monomorphic PVCs but the site of origin must first be identified. This process often requires long, invasive mapping procedures using activation mapping and pace mapping techniques.

Non-invasive localization of the site of origin could provide valuable pre-procedural insight to reduce operating times and improve outcomes. Electrocardiographic imaging (ECGI) is a promising tool to achieve this goal by reconstructing a high-resolution projection of cardiac electrical events from non-invasively measured body surface potentials (Cluitmans et al., 2022).

As a natural consequence of the goal of identifying PVCs, they have become one of the most commonly targeted pathologies for ECGI validation (Barr et al., 1977; Oster et al., 1998; Han et al., 2011; Sapp et al., 2012; van Dam et al., 2013; Cluitmans et al., 2017; Wissner et al., 2017; Bear et al., 2018b; Graham et al., 2019; Svehlikova et al., 2022). However, the results of validation studies show a surprising range of reconstruction accuracy for the site of origin, from 5 to 50 mm in healthy hearts (Oster et al., 1998; Han et al., 2011; Cluitmans et al., 2017; Bear et al., 2018b; Svehlikova et al., 2022), even when using similar ECGI methods. There is also a significant reduction in accuracy seen in the presence of structural heart disease (Sapp et al., 2012; Graham et al., 2019), suggesting an underlying pathology could also impact accuracy. To both control the rate of sample beats and dictate their site of origin, the majority of these studies rely on ventricular stimulation to mimic PVCs. However, it remains unclear if stimulation produces activation sequences that mimic those from spontaneous PVCs. Differences in the activation sequence could arise in physiologically healthy cases and even more likely in the context of pathophysiological changes to the heart tissue. Furthermore, such complicated spontaneous PVC morphologies as the R-on-T phenomenon have never been reported in ECGI validation studies.

Difficulty in validating ECGI reconstruction of PVCs also stems from a lack of detailed knowledge of key parameters. For spontaneous PVCs in a clinical setting, the successful site of ablation provides the only ground truth data available to validate ECGI (van Dam et al., 2013; Svehlikova et al., 2022). Experimental studies can provide slightly more detailed forms of validation for the location of spontaneous PVCs by recording electrical signals on and throughout the heart; however, in such cases, it can be challenging to reliably induce spontaneous PVCs, and thus, ventricular stimulation is often used as a surrogate to mimic spontaneous PVCs (Bergquist et al., 2021). The diversity in accuracy may also be due to the different methods used to localize the site of origin, including direct analysis of the reconstructed epicardial potentials or using post-processing and analyzing activation time (AT) maps. To date, no studies have compared these different methods or their accuracy in localizing focal ventricular activity in either measured or reconstructed epicardial signals.

The objective of this study was to explore the differences in accuracy of an epicardial potential-based formulation of ECGI when reconstructing spontaneous PVCs as compared to ventricular-paced beats. We also explored the impact of pathophysiological perturbation on ECGI reconstruction accuracy in the context of local hyperkalemia, a major contributor to the electrical changes seen in acute myocardial ischemia. Finally, we evaluated the different methods to localize the site of origin of ectopic beats using ECGI, including the epicardial breakthrough site and the depth of the ectopy in the myocardium.

## 2 Methods

### 2.1 Experimental data

Animal experiments were carried out in accordance with institutional guidelines and the recommendations of the Directive 2010/63/EU of the European Parliament on the protection of animals used for scientific purposes and approved by the local ethical committee of Bordeaux CEEA50.

**FIGURE 1:**
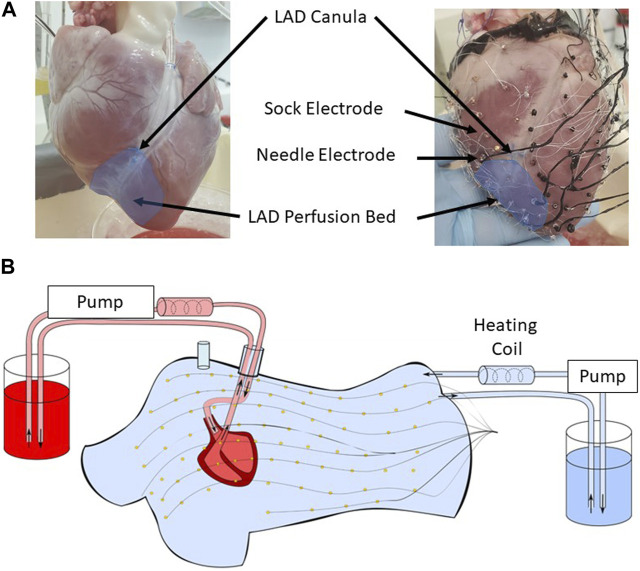
**(A)** The lower half of the left anterior descending artery (LAD) was cannulated on a separate perfusion (left). An epicardial electrode sock (128 electrodes) was attached to the ventricles, and nine intramural plunge needles were inserted into the myocardium to record the cardiac electrical activity (right). **(B)** The heart was placed in a human torso-shaped tank embedded with 256 electrodes. The heart was perfused with a 1:2 mixture of blood and Tyrode’s solution (red), and the tank was filled with an electrolytic solution at 500 ohm cm (blue), both kept at 37 °C with heating coils.

The experimental preparation consisted of an isolated and perfused animal heart suspended in an *ex vivo* human-shaped torso tank, a model previously described by Bear et al. (2018a) and Bear et al. (2021). The hearts were excised from male pigs (*n* = 3; 35–37 kg) and transported in an ice-cold cardioplegic solution to the laboratory. The hearts were then suspended and perfused in Langendorff mode with a 1:2 mixture of blood and Tyrode’s solution, oxygenated with 95%/5% O_2_/CO_2_ (pH 7.4, at 37 °C). The left anterior descending artery (LAD) was cannulated half-way down the ventricle and perfused via a separate line from the aorta (Figure 1A). A custom epicardial electrode sock (128 electrodes, 8 ± 2 mm spacing) was attached to the ventricles, and nine intramural plunge needles (10 electrodes each, 2 mm spacing) were inserted into the myocardium as described by Zenger et al. (2020) and demonstrated in Figure 1A. The instrumented heart was then placed in a human torso-shaped tank embedded with 256 electrodes (30–60 mm spacing) and filled with an electrolytic solution with the resistivity set to 500 Ω cm (Figure 1B). Local hyperkalemia was induced through perfusion of a high-K^+^ solution (8 mM) into the LAD, modeling one of the major ionic changes resulting from acute ischemia (Morena et al., 1980). Unipolar potentials from the torso, sock, and needle electrodes were simultaneously recorded (BioSemi, the Netherlands) following bipolar pacing from various needle electrodes at different depths and in sinus rhythm, during which spontaneous PVCs occurred. Recordings were repeated at baseline and at high K^+^. Bipolar pacing used two neighboring electrodes and was performed by 2 ms pulses at 2.5 Hz with constant current amplitudes approximately 1.5 x the diastolic threshold. High K^+^ perfusion was performed for 15 min in order for the electrical effects to stabilize before pacing was performed. Signals were sampled at 2048 Hz and referenced to the Wilson central terminal. Also, 3D-rotational fluoroscopy (Artis, Siemens) was used to obtain subject-specific electrode locations and coronary positions at the end of each experiment.

### 2.2 Signal processing and inverse methods

Signal processing was necessary before applying ECGI to reconstruct epicardial potentials. First, PVCs were manually identified by their different QRS morphologies from those in sinus rhythm. PVCs were defined as being R-on-T if they occurred simultaneously with the T-wave of the preceding sinus beat. Individual PVC and stimulated beats were segmented manually, and baseline drift was removed using previously described methods (Rodenhauser et al., 2018). Intramural stimulated beats using the needle electrodes were identified as either sub-epicardial or sub-endocardial depending on the surface the electrodes were closer to. Broken electrodes were identified manually and removed from the analysis (28–33 sock electrodes and 9–12 torso electrodes). Epicardial surface meshes were created for each experiment from sock electrode locations by manual triangulation of the electrode locations followed by capping the base of the heart to produce a closed epicardial mesh (146–179 nodes, 2–31 mm internode spacing). The torso surface mesh was created from triangulated torso electrode locations and was the same for all experiments (256 nodes, 30–60 mm internode spacing).

Based on the tank and heart geometries, a homogeneous forward model was computed between the epicardial and torso surface meshes using the boundary element method (BEM) (Barr et al., 1977). Epicardial potentials were reconstructed from recorded tank potentials using Tikhonov zero-order regularization (Tikhonov and Arsenin, 1977) using the median lambda calculated over the QRS with the L-curve method (Hansen et al., 1993). ATs were derived from ECGI and sock EGMs as the time of the minimum dV/dt. For ECGI, the ATs were then spatially smoothed by combining them with an estimate of the local spatial gradient (Cluitmans et al., 2022), a method well known to increase the accuracy of the reconstructed ATs over the traditional temporal-only method that are more prone to lines of false conduction block and poor estimates for ectopic localization (Cluitmans et al., 2017; Duchateau et al., 2019; Schuler et al., 2021).

### 2.3 Evaluation methods

Quantitative evaluation of reconstruction accuracy was performed by comparing ECGI reconstructions to corresponding sock-recorded electrograms (EGMs), potential maps, and ATs. The following describes the metrics used to compare each of these features.

#### 2.3.1 Unipolar EGM morphology

Recorded and reconstructed EGMs were compared using Pearson’s correlation coefficient (CC) between EGMs at each electrode and the difference in the peak-to-peak amplitude (Pk–Pk) over the QRS. The mean of each metric was computed over 1) all electrodes and 2) the first 10% of electrodes to activate, defined as the early activating (EA) electrodes.

#### 2.3.2 Potential maps

For each time step, ECGI and recorded potential maps were first compared using the CC at each point in time. The center of the negative region of epicardial potential maps at a time point early in the QRS has previously been used with ECGI to estimate the epicardial breakthrough sites (Oster et al., 1997). In order to enhance these negative potentials, the broken electrodes identified in recorded potential maps were first interpolated using a Laplacian spatial filter (Oostendorp et al., 1989; Bergquist et al., 2019) implemented in the open-source SCIRun package (https://www.sci.utah.edu/cibc-software/scirun.html). All of the positive potentials were set to zero with a threshold of >−5 % of Pk–Pk magnitude to avoid noise. These maps were defined as negative potential enhanced maps (NPEMs). The recorded and reconstructed NPEMs were compared using five metrics:

1 The CC at each point in time
2 The weighted under-estimation indicator (WUI) detecting the percentage of recorded negative potentials that are not detected
3 The weighted overestimation indicator (WOI) detecting the percentage of the misjudged ECGI negative potentials
4 The Euclidean distance between the centers of the largest measured and ECGI negative potential regions

The WOI and WUI were previously used to evaluate the accuracy of ECGI for ischemia detection (Wang et al., 2010) and singularity point detection in atrial fibrillation (Figuera et al., 2016). Here, the formulas have been weighted by the values in the region of interest as in the work of Figuera et al. (2016) and in this case by the potential value *Ve* (*t*, *n*) at each node *n*, defined as

$$WUI\%\left(t\right)=100\frac{\sum_{n\in FN}Ve\left(t,n\right)}{\sum_{n\in FN}Ve\left(t,n\right)+\sum_{n\in TP}Ve\left(t,n\right)},$$ (1)

$$WOI\%\left(t\right)=100\frac{\sum_{n\in FP}Ve\left(t,n\right)}{\sum_{n\in FP}Ve\left(t,n\right)+\sum_{n\in TP}Ve\left(t,n\right)},$$ (2)

where FN (false negative) is the set of nodes belonging to the recorded negative potential region but not to the ECGI region, TP (true positive) is the set of nodes in both the recorded and ECGI negative potential regions, and FP (false positive) is the nodes belonging to the ECGI negative potential region but not to the recorded. Here, the negative potential regions are any nodes not set to 0.

The center of the negative potential regions was then defined as the weighted mean of the largest group of connecting nodes with negative potentials, orthogonally projected to the epicardial surface, where the nodes are weighted by their negative potential magnitude. The Euclidean distance was also calculated between the center of the sock negative region and the earliest activation site defined in the following section. The mean of each of the four metrics was taken over: 1) the full QRS interval and 2) the first 10% of the QRS interval defined as the early activation period. Finally, the temporal delay in the first appearance of a negative potential (delay) was also computed between recorded and ECGI maps.

#### 2.3.3 AT maps

The epicardial breakthrough site was defined from measured and ECGI ATs as the center of the area covered by the first 10% of total epicardial AT. In the case of two disconnected early activating regions, we used only the largest contiguous region. Using simulated data, Rupp et al. (2020) demonstrated a strong linear relationship between the depth within the myocardium of the ectopic origin and the area covered by the first 10% of total epicardial AT (Area_10*% AT*_) (Rupp et al., 2020). We, therefore, calculated the Area_10*% AT*_ by summing the area of the triangles in which all three nodes were a part of the earliest 10% activated.

ATs computed with ECGI were compared to those recorded using CC and the mean absolute error (MAE). Localization error (LE) was computed between ECGI and measured epicardial breakthroughs using the Euclidean distance. Recorded and ECGI Area_10*% AT*_ during sub-epicardial and sub-endocardial pacing were compared.

#### 2.3.4 Statistical methods

The accuracy of ECGI was first evaluated using PVCs and pacing data at baseline. The impact of physiological condition on ECGI accuracy was then evaluated by comparing the previous baseline ectopy data (PVC and stimulated) to the ectopies measured during high K^+^ perfusion. In addition, reconstruction accuracy of PVCs occurring on the T-wave (R-on-T) at baseline and at high K^+^ perfusion was assessed. Statistical analysis was conducted using GraphPad Prism 9.5.1. A two-way ANOVA was used to compare accuracy between beat types (PVC, sub-epicardial, or sub-endocardial pacing) and physiological condition (baseline or high K^+^) with p < 0.05 defined as significant. Data in tables and error bars in figures are expressed as mean ± standard deviation unless otherwise stated. In figures, the symbols *, **, and*** indicate *p*-values <0.05,< 0.01 and < 0.001.

## 3 Results

In total, for the three experiments, the number of paced (sub-epicardial vs. sub-endocardial) and spontaneous PVC (standard vs. R-on-T) beats used in the following analysis is presented in Table 1 at baseline and during high K^+^ perfusion. In general, more ventricular sites were successfully paced at baseline, while more spontaneous PVCs were present during high K^+^ perfusion. Spontaneous VF occurred in one of the experiments during the 15 min high K^+^ induction period. No spontaneous VF was seen in any heart at baseline.

**TABLE 1:** Summary of the number of cardiac beats used for analysis.

	Baseline	High K+
**Total # of pacing beat**	**30**	**15**
Sub-epicardial pacing	14	4
Sub-endocardial pacing	16	11
**Total # of PVCs**	**22**	**34**
Standard PVCs	26	38
R-on-T PVCs	4	4

### 3.1 Ectopy reconstruction in healthy hearts

#### 3.1.1 Electrograms

**FIGURE 2:**
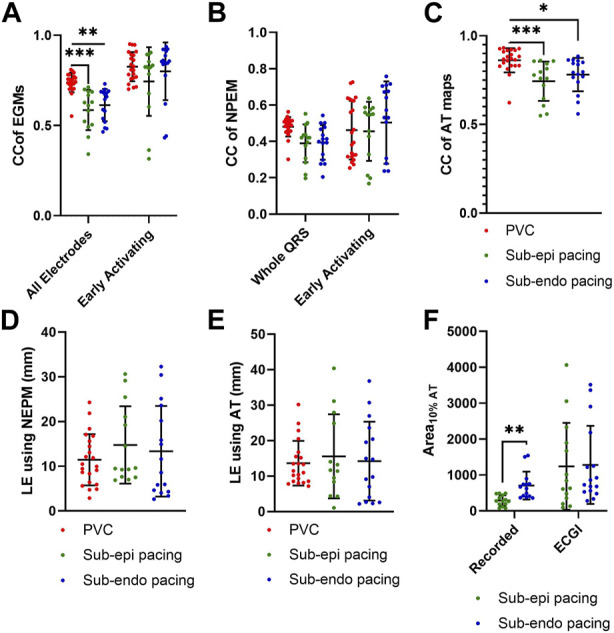
**(A–C)** Recorded (black) and ECGI reconstructed (blue) EGMs at baseline for a representative **(A)** PVC, **(B)** sub-epicardial pacing, and **(C)** sub-endocardial pacing beat. CC between ECGI and recorded EGMs are presented in each EGM. **(D–F)** Corresponding AT map and electrode **(E)** locations for each EGM plots for each representative beat. The red zone represents the QRS interval over which the CC was computed.

Epicardial EGMs reconstructed using ECGI were compared to those recorded with the sock at baseline. Figure 2 presents recorded (black) and ECGI reconstructed (blue) EGMs close to the origin of ectopy for a representative A) PVC, B) sub-epicardial paced, and C) sub-endocardial paced beats. CC between reconstructed and recorded EGMs are presented in each plot. The corresponding AT map and electrode locations for each EGM plot are presented for each representative beat (D, E, and F).

In general, the reconstructed EGMs had a similar morphology to those recorded, although they were typically smaller in amplitude. However, for all three representative beats, a temporal delay could often be seen in the reconstructed EGMs that activated earliest, with the intrinsic deflection occurring later and the QRS upstroke earlier (Figure 2A: E1–E3, B: E1–E3, and C: E3 and E4). This delay tended to be longer for the sub-epicardial paced beats and for certain PVCs and could negatively impact the CC despite global QRS morphology corresponding to the ground truth. In several cases, detailed morphological features, such as R-waves (Figure 2A: E3, E4, B: E4 and E6, and C: E6) and fractionation (Figure 2A: E2 and E5), were displaced or missing which also impacted CC values. This was again more common during sub-epicardial than sub-endocardial pacing. Slightly later EGMs appeared to be well aligned temporally, as reflected by higher CC (Figure 2B: E6 and C:E6).

**FIGURE 3:**
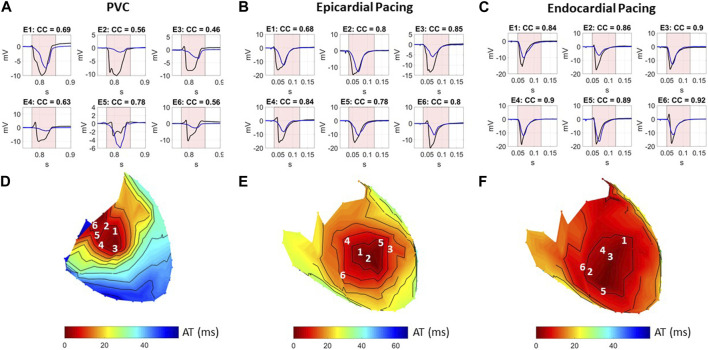
Boxplots of CC between recorded and ECGI reconstructed **(A)** EGMs, **(B)** NPEM, and **(C)** AT maps; LE of epicardial breakthrough sites using **(D)** NPEM, **(E)** AT maps, and **(F)** Area_10*% AT*_. Values are presented for PVCs (red), sub-epicardial pacing (green), and sub-endocardial pacing (blue) beats.

Figure 3A presents the CC between EGMs for all standard PVC and paced beats, averaged over either all electrodes or only the early activation electrodes. Globally, the morphology of EGMs from spontaneous PVCs, as measured by CC were better reconstructed than those during sub-endocardial (p < 0.001) and sub-epicardial pacing (p < 0.01). The early activation sites produced higher CC than the average over all electrodes for all beat types (p < 0.01). Likewise, the variability in accuracy in early activation sites was higher during pacing than spontaneous PVCs (SD = 0.16–0.19 vs. 0.08). ECGI tended to slightly underestimate the QRS amplitude as measured by the Pk–Pk amplitude differences (Table 2) with no significant difference between beat types or between all and only the early activation electrodes.

**TABLE 2:** Mean ± standard deviations of the different metrics used to evaluate ECGI electrograms, potential maps (PMs), negative potential enhance maps, and activation times at baseline and high K+. EGMs were averaged over all (All) or the early activating electrodes. PM were averaged over all (All) or the early activation portion of the QRS. * represents a significant difference with equivalent (baseline or high K+) PVC results. ^**‡**^ represents a significant difference with equivalent (baseline or high K+) sub-epicardial pacing results. ^**†**^ represents a significant difference with the equivalent beat type (PVC, sub-epicardial, or sub-endocardial) at baseline.

	Isolated PVC		Sub-epicardial pacing		Sub-endocardial pacing	
	Baseline	High K+	Baseline	High K+	Baseline	High K+
CC-EGM-All	0.74 ± 0.05	0.68 ± 0.10^**†**^	0.59 ± 0.11*	0.58 ± 0.11*	0.61 ± 0.09*	0.65 ± 0.10
CC-EGM-EA	0.83 ± 0.08	0.77 ± 0.17	0.74 ± 0.19	0.81 ± 0.08	0.80 ± 0.16	0.80 ± 0.06
Pk–Pk-All (mV)	4.9 ± 1.0	5.1 ± 1.0	4.2 ± 1.4	4.7 ± 1.2	4.4 ± 1.6	4.8 ± 1.2
Pk–Pk-EA (mV)	4.5 ± 2.2	4.5 ± 2.2	4.0 ± 3.9	4.1 ± 5.2	4.3 ± 3.2	5.4 ± 4.0
CC-PM-All	0.61 ± 0.06	0.62 ± 0.14	0.50 ± 0.12*	0.60 ± 0.19	0.52 ± 0.11*	0.70 ± 0.18^**†**^
CC-PM-EA	0.48 ± 0.15	0.43 ± 0.13	0.47 ± 0.18	0.38 ± 0.24	0.52 ± 0.23	0.53 ± 0.23
CC-NPEM-All	0.48 ± 0.05	0.39 ± 0.07^**†**^	0.39 ± 0.10*	0.45 ± 0.14	0.40 ± 0.10*	0.49 ± 0.11* ^**†**^
CC-NPEM-EA	0.46 ± 0.16	0.45 ± 0.12	0.46 ± 0.16	0.46 ± 0.19	0.50 ± 0.23	0.53 ± 0.17
WUI-EA	48 ± 25	63 ± 14^**†**^	41 ± 25	35 ± 40*	37 ± 30	30 ± 25*
WOI-EA	43 ± 18	48 ± 16	57 ± 18*	59 ± 16	49 ± 22	45 ± 20
Delay (ms)	6.0 ± 6.2	14.9 ± 14.2^**†**^	5.5 ± 1.9	9.2 ± 5.6	2.8 ± 2.6^**‡**^	2.7 ± 3.7*
LE-NPEM (mm)	11.0 ± 5.6	13.3 ± 5.5	14.8 ± 8.6	13.2 ± 12.8	13.3 ± 10.1	12.4 ± 10.3
CC-AT	0.86 ± 0.07	0.70 ± 0.12^**†**^	0.74 ± 0.11*	0.38 ± 0.34* ^**†**^	0.78 ± 0.09*	0.67 ± 0.11^**‡†**^
MAE-AT (ms)	6.4 ± 1.5	9.8 ± 2.0^**†**^	8.3 ± 1.7*	15.7 ± 2.5* ^**†**^	7.8 ± 1.8*	11.6 ± 2.3* ^**‡†**^
LE-AT (mm)	13.7 ± 6.3	17.4 ± 9.8	15.6 ± 11.9	14.6 ± 6.3	14.2 ± 11.1	19.8 ± 11.9

#### 3.1.2 Potential maps

**FIGURE 4:**
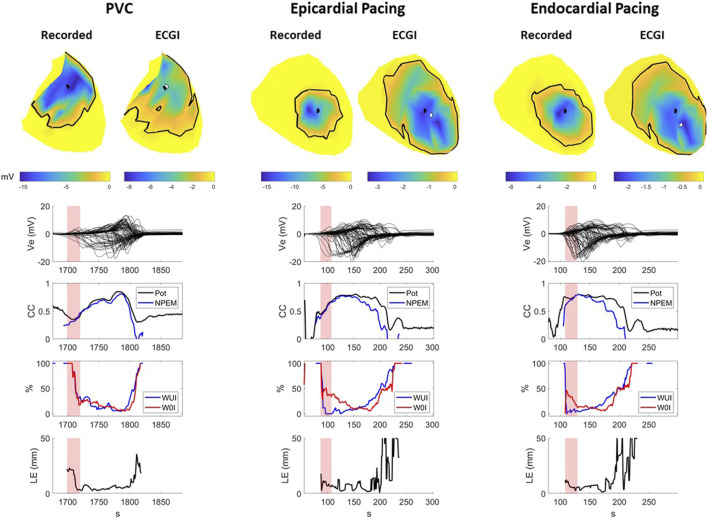
Recorded and ECGI reconstructed potential maps during early activation at baseline for a representative PVC (left), sub-epicardial pacing (middle), and sub-endocardial (right) pacing beat. The black and white dots represent the recorded and ECGI epicardial breakthrough, respectively, computed using the center of the negative potential region. The black isochrone outlines the negative potential region. For each potential map, the corresponding sock electrograms (top), CC of potential maps (pot) and NPEM (second row), the WOI and WUI (third row), and localization error (bottom row) are presented for each moment in time. The red zone represents the EA period for each beat used for averaging the different metrics.

Figure 4 presents A) recorded and reconstructed NPEM for a representative PVC (left), those paced from the sub-epicardium (middle) and sub-endocardium (right). CC between the reconstructed and recorded potentials maps and NPEM are presented (third row), the WUI and WOI metrics (fourth row), and the localization error between the centers of the recorded and reconstructed negative potential regions (last row).

The recorded negative potential area appeared before those reconstructed with ECGI, with a greater delay the sub-epicardial paced beat than the spontaneous PVC or sub-endocardial paced beats. The recorded NPEM demonstrated negative regions with smooth borders, where the most negative potentials were not necessarily at the center but could occur at the edge of the negative region (see PVC example, Figure 4). ECGI on the other hand tended to produce negative regions with jagged borders and the most negative point near the center. Near the beginning of activation, the negative regions were misplaced by ECGI compared to the recordings, giving low CC (below 0.5) for both potential maps and NPEM. Once the negative region grew big enough later in activation, overlap of the recorded and ECGI regions occurred and CC increased. The reconstructed negative-potential zones also tended to be largely overestimated in size compared to those recorded, as seen by increased WUI and WOI values, and depending on the time frame, upwards of 20–30 mm in localization errors were seen between their centers. There were no obvious differences in reconstruction accuracy among spontaneous PVCs and paced beats, either visually or according to any metrics.

Across all beats, ECGI demonstrated a delay of 0–6 ms when compared to recorded potential maps (Table 2). This delay was significantly longer in sub-epicardial than sub-endocardial paced beats (*p* = 0.01). Despite the longer delay for NPEM to start, there was no difference in their CC during the early activation phase (Figure 3B). Likewise, there was no significant difference (p > 0.46) seen in the ECGI localized origin of these ectopies using NPEM (Figure 3D). In early activation, the reconstructed negative potential area covered the recorded negative potentials to a similar extent for all beat types (p > 0.1 for WUI) though the reconstructed region was significantly larger than it should be, in particular during sub-epicardial pacing (*p* = 0.02 comparing PVC to sub-epicardial WUI). Interestingly, the NPEM produced CC that were slightly lower than those using the full potential map (Table 2), suggesting the negative potentials may not be captured as well as positive potentials.

#### 3.1.3 Activation maps

**FIGURE 5:**
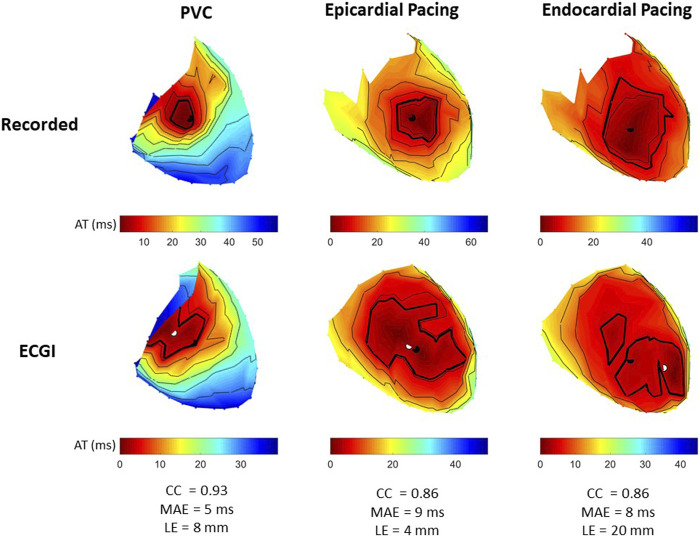
Recorded and ECGI reconstructed AT maps at baseline for a representative PVC (left), sub-epicardial pacing (middle), and sub-endocardial (right) pacing beat. Isochrones are displayed every 5 ms, with the 10% activation line in bold. The black and white dots represent the recorded and ECGI epicardial breakthrough, respectively, computed using the earliest AT.

Figure 5 presents recorded (top row) and reconstructed (bottom row) AT maps for a representative PVC (left), sub-epicardial paced (middle), and sub-endocardial paced (right) beat. The epicardial breakthrough site as detected by the earliest AT is represented by a black sphere for recorded and a white sphere for reconstructed signals. In each AT map, the isocontours were placed at 5 ms intervals, with a thick isoline representing the border of the region used to compute Area_10*% AT*_ (the first 10% of total epicardial activation).

From recorded AT maps, we see the activation pattern from the PVC was more complex than that seen with pacing; that is, a region of very slow conduction occurred near the epicardial breakthrough for the PVC beat, while both paced beats showed smooth activation wavefronts spreading from the breakthrough. The Area_10*% AT*_ was smaller for sub-epicardial than for sub-endocardial paced beats in recorded maps.

Despite the reconstructed AT map missing the slow conduction for the PVC beat, the CC demonstrated there had a strong correspondence to the recorded propagation pattern. Similar to the PVC, ECGI did not capture the slow activation near the epicardial breakthrough site during sub-epicardial pacing, rather we saw a large area activating nearly simultaneously. This meant that the Area_10*% AT*_ during sub-epicardial pacing was of a similar size to seen with ECGI for sub-endocardial paced beat, preventing us for determining the myocardial depth of the pacing electrodes. Furthermore, although most paced beats were similar to sub-epicardial paced beat, in some cases (as in the sub-endocardial paced beat), ECGI produced multiple epicardial breakthroughs as opposed to a single breakthrough as seen in the recorded maps. In this case, the earliest activation site did not correspond to the true epicardial breakthrough, resulting in a larger localization error. For all three beat morphologies (spontaneous PVC, sub-endocardial, and sub-epicardial paced beats), the total activation time reconstructed with ECGI was shorter than recorded.

Figure 3C presents the CC between AT maps for all PVC and paced beats. AT maps were better reconstructed for PVCs than during sub-epicardial (p < 0.001) and sub-endocardial pacing (p < 0.05). The timing of these maps was likewise better for PVCs (see MAE Table 2). Although this improved accuracy, there was no significant difference in the LE between the different beat types (Figure 3E). Figure 3F presents the Area_10*% AT*_ computed during sub-epicardial and sub-endocardial pacing for recorded and reconstructed AT maps. From recorded AT maps, sub-endocardial pacing resulted in an average Area_10*% AT*_ more than twice as large as for sub-epicardial pacing (p < 0.001). The reconstructed Area_10*% AT*_ was overestimated for sub-epicardial paced beats when compared to recorded values (p < 0.01), and there was no difference in Area_10*% AT*_ between sub-epicardial and sub-endocardial paced beats (*p* = 0.93).

### 3.2 Impact of the physiological phenomenon

#### 3.2.1 High K^+^ perfusion

The impact of hyperkalemia on reconstruction accuracy was evaluated by comparing the various evaluation metrics at baseline and during high K^+^ perfusion for PVC and paced beats. Table 2 presents the mean ± standard deviations for each of these metrics at baseline and high K^+^.

**FIGURE 6:**
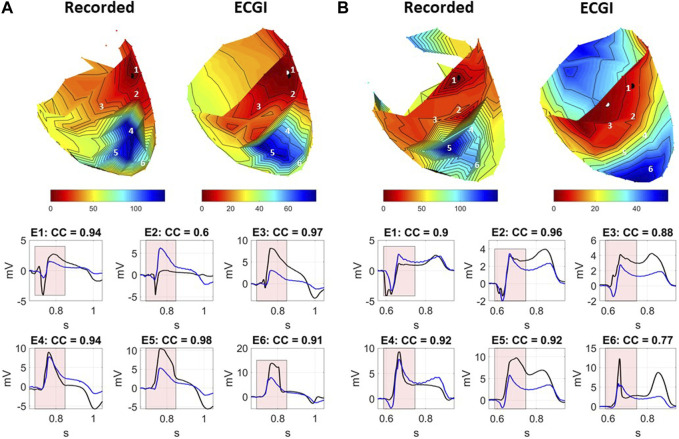
Recorded and ECGI reconstructed AT maps (top) for **(A)** a well-reconstructed and **(B)** a poorly reconstructed PVC during high K+ perfusion. Isochrones are displayed every 5 ms. The black and white dots represent the recorded and ECGI epicardial breakthrough, respectively, computed using the earliest AT. Numbers indicate the locations for the recorded (black) and ECGI reconstructed (blue) EGMs presented as follows. CC between ECGI and recorded EGMs are presented in each EGM. The red zone represents the QRS interval over which the CC was computed.

Figure 6 presents recorded (black) and reconstructed (blue) electrograms and activation maps for two example PVCs during high K^+^ perfusion for the same experiment. For each example, electrode locations are labeled on the equivalent recorded and ECGI reconstructed AT maps. PVC A is an example in which ECGI very accurately captured the PVC origin as well as the resulting AT map including the late region of activation within the high K^+^ perfusion bed (CC = 0.91, LE = 3 mm). On the other hand, PVC B is one of the least accurate examples (CC = 0.60), with a larger error in PVC origin localization (LE = 17 mm) and a clear shift in the late activation region. This shift is due to a misplacement of the AT on EGMs presenting with two down-strokes (e.g., electrodes 4 and 5), which were not present in PVC A.

Overall, for both examples, the general morphology of the QRS was captured near the site of origin and in the K^+^ perfusion bed, including the ST segment elevation. However, as was seen at baseline (Figure 2), a temporal delay in the intrinsic deflection was seen in reconstructed EGMs near the site of origin, even in the well-reconstructed example (electrode 1 for both PVCs). The sharp intrinsic deflection in the electrograms near the K^+^ perfusion bed border zone was often smoothed out by ECGI (electrode 6 in both examples). Furthermore, fractionation was intensified in electrodes near the perfusion bed which ECGI failed to capture (electrodes 2 and 3 in example B).

Over all beats, the mean CC averaged over all electrodes was reduced with K^+^ perfusion for PVCs (*p* = 0.04) but not for paced beats (*p* = 0.30 and *p* = 0.95 for sub-endocardial and sub-epicardial pacing, respectively). The early activation sites likewise showed a non-significant decrease in mean CC for PVCs (*p* = 0.13). There was no significant difference in Pk–Pk amplitude differences with high K^+^, though average values tended to be higher than at baseline.

The mean CC of potential maps averaged over the whole QRS with K^+^ perfusion tended to be higher across all three beat types, but only significantly for sub-endocardial paced beats (*p* = 0.01). The CC of potential maps and NPEM over the early activation period of the QRS did not change significantly. There was no change in WOI (p > 0.30), whereas WUI were higher for PVCs (*p* = 0.02) demonstrating that the recorded NPEMs were less well captured by ECGI with high K^+^ than at baseline. There was also a large increase in the Delay with ECGI for PVCs (p < 0.001), but not for sub-epicardial (*p* = 0.47) or sub-endocardial pacing (*p* = 0.96). There was no significant change in LE using NPEM (p > 0.26).

The accuracy of the reconstructed AT map was also worse for both PVCs and paced beats in terms of CC and MAE (p < 0.001). There was no significant change in LE using AT, though for PVC and sub-endocardial pacing the mean LE increased, as with baseline recordings, sub-endocardial pacing resulted in larger Area_10*% AT*_ than sub-epicardial pacing (p < 0.01), with no change in area compared to baseline (p > 0.19). Likewise, ECGI overestimated the size of the region during sub-epicardial pacing and was unable to demonstrate a difference between sub-epicardial and sub-endocardial pacing.

#### 3.2.2 R-on-T PVCs

**FIGURE 7:**
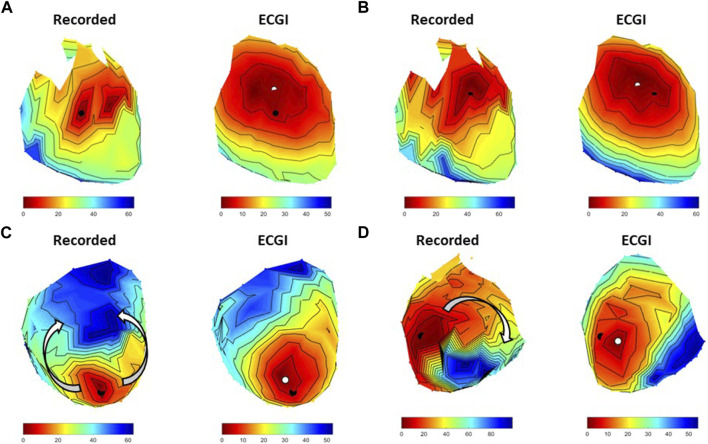
Four examples recorded and ECGI reconstructed AT maps during **(A)** an isolated PVC at baseline, **(B)** an R-on-T PVC from the same origin as A at baseline, **(C)** a second R-on-T PVC at baseline, and **(D)** an R-on-T PVC during high K+ perfusion. The black and white dots represent the recorded and ECGI computed epicardial breakthrough, respectively.

While the majority of spontaneous PVCs recorded during these experiments occurred after the T-wave, eight R-on-T PVCs were also recorded (four at baseline and four during high K^+^ perfusion) and were not included in the previous analysis. Figure 7 presents AT maps for three R-on-T PVCs alongside that from standard PVC at baseline.

The R-on-T PVC at baseline in Figure 7B had a similar epicardial breakthrough location to the standard PVC in Figure 7A. As can be seen, despite occurring on the T-wave, the recorded activation wavefront followed a similar trajectory to the standard PVC, albeit taking longer for the whole heart to activate. ECGI captured the general trajectory well for both isolated (CC = 0.86) and R-on-T (CC = 0.91), as well as the slower conduction velocity in the R-on-T example, though the reconstructed wavefront was smoother in both cases than recorded. Figure 7C presents an alternative R-on-T PVC at baseline. In this case, the epicardial breakthrough of the PVC occurred near an area of late repolarization from the preceding beat, causing the recorded activation wavefront to block and circumnavigate the region as indicated by the arrows. While ECGI captured the epicardial breakthrough fairly accurately (LE = 8.2 mm), the activation block was not captured and rather a slow smooth activation from the site of origin was reconstructed, reflected by the lower CC for this map (CC = 0.68). Finally, Figure 7D presents an R-on-T PVC during high K^+^ perfusion. Here, the onset was close to the K^+^ perfusion zone, which blocked the wavefront resulting in a non-sustained reentrant circuit. In this case, ECGI captured this block but the pathway of activation was altered and the total activation time was substantially shorter than recorded (CC = 0.65).

## 4 Discussion

The clinical use of ECGI has attracted considerable interest in recent years, in particular to identify the origin of PVCs to aid in ablation therapy (Parreira et al., 2020; Nguyen et al., 2021). Though numerous studies have targeted PVC localization for ECGI validation, the majority have relied upon experimental or clinical data using ventricular stimulation to model PVCs. Furthermore, these data are most often produced using healthy hearts.

Our study is the first to compare the reconstruction accuracy of ECGI for natural PVCs in ventricular stimulation using both healthy hearts and those with regional hyperkalemia. Here, we demonstrate that

1 Subtle differences exist between the use of spontaneous PVCs and ventricular pacing for ECGI validation
2 Localizing the early negative region in potential maps is the preferred method for identifying epicardial breakthroughs
3 Contemporary ECGI formulations cannot distinguish epicardial from endocardial sources using a potential-based approach
4 An underlying pathology can impact accuracy of ectopy localization, particularly when the origin is close the abnormal tissue

### 4.1 Spontaneous PVC vs. ventricular pacing for ECGI validation

In general, only subtle differences existed in ECGI reconstruction accuracy between spontaneous PVC and pacing data. Unexpectedly, we found PVCs to be slightly better reconstructed than paced beats; that is, the CC of electrograms, potential maps, and AT maps was significantly better at baseline for spontaneous PVCs than for either sub-epicardial or sub-endocardial paced beats (see Table 2). While statistically, there were no differences between pacing and spontaneous PVCs in the early activation phases, further analysis using WOI and WUI demonstrated that the negative region was better captured in PVCs than with pacing. This subtle difference was then seen in the overall higher LE for pacing data, although the number of data points is insufficient to make this significant.

In previous studies, LE of ventricular pacing was significantly greater when the stimulus to QRS interval was longer (Graham et al., 2019). In this study, it may be the case that pacing activated a lower initial volume of tissue than spontaneous PVCs, therefore taking longer for main wavefront propagation to take place (the equivalent of having a longer stimulus to QRS interval). Altering the current, pulse duration [or potentially the frequency of pacing (Perez Alday et al., 2019)] would likely alter this initial volume of tissue activated and could impact ECGI reconstruction accuracy particularly in the early phase of activation. However, the mechanism by which this impacts reconstruction accuracy remain unclear.

Despite the subtle differences seen between pacing and PVCs, the benefits of using ventricular pacing cannot be ignored. Having the accurate location of the stimulus site based on the pacing electrode location, including myocardial depth, is very important if testing methods are required to determine epicardial vs. endocardial origin. However, while pacing data still provide meaningful information for ECGI evaluations, we believe that PVCs should be used with preference when available. More validation studies are also needed using both R-on-T PVCs, particularly when encountering obstacles (functional or anatomical) which cause line of block and re-entry (see Figure 7). As these are hard to record, a first step would be to use ventricular pacing on the T-wave of sinus beats.

### 4.2 Impact of physiological abnormality

In the presence of local hyperkalemia, we saw little significant change in the reconstruction of EGMs, but a large reduction in accuracy in AT maps. Inaccuracies appeared to be more prominent near and in the perfusion bed. From EGMs, ECGI failed to capture important but subtle fractionation in the high K^+^ regions (see Figure 6) which likely resulted in false identification of the AT markers. Though LE for paced beats and natural PVCs tended to be elevated in hyperkalemia, the changes were not significant (see Table 2).

In a previous study, Sapp et al. (2012) used epicardial catheter mapping in four patients with myocardial infarction to demonstrate that pacing site localization is dependent on the proximity to the scar border. We assessed this phenomenon by separating the PVCs into those close to ( < 25 mm) and far from ( >= 25 mm) the border of the perfusion bed. As in the work of Sapp et al., LE was increased when foci lay closer to the perfusion bed for both NPEM and AT methods (see Supplementary Figure S1).

Based on these results and the minimal global changes in accuracy we saw with high K^+^ perfusion in this study, we suspect that the accuracy of ECGI reconstructions is only changed near the high K^+^ region with the accuracy of reconstruction remaining as good as in a healthy heart in the remainder of the heart. This highlights the importance of validating ECGI using pathophysiological models and potentially the need to incorporate the electrical changes of these models into the forward model to improve ECGI accuracy near the abnormality.

### 4.3 Method to localize ectopic origins

Two main methods exist to determine the epicardial site of origin for PVC or pacing beats using ECGI; finding the center of the region of negativity in potential maps early in the QRS (Oster et al., 1997; Wang et al., 2011; Sapp et al., 2012) or finding the earliest site of activation (Cluitmans et al., 2017; Bear et al., 2018b; Graham et al., 2019). To our knowledge, this is the first study to evaluate the accuracy of both methods for ECGI.

While we found no significant difference in the LE when using AT or the NPEM approach, the AT method produced consistently higher mean LE compared to the NPEM and larger outliers. In this study, both methods were dependent on automated methods whose errors could potentially be improved using manual selection. However, in the case of AT maps, this is two-fold, requiring a manual check on the AT annotations and assessing EGMs in the case of multiple breakthrough sites to determine the true origin. For the NPEM approach, in the authors’ perspective, a manual check is much simpler, only requiring one to look at potential maps over multiple time instances during early activation to find the true center of the early negative region. We, therefore, believe that using potential maps should be the preferred approach to localize ectopic origins for epicardial potential-based ECGI. For transmembrane voltage-based ECGI or activation/recovery-based ECGI, the NPEM method is no longer valid, and therefore, localization must be based on AT.

A key aspect for both methods is the accuracy with which ECGI can capture the intrinsic deflection in the early activating regions. From EGMs, we found ECGI produced an important temporal lag in the onset of the intrinsic deflection (Figures 2, 6). This is likely due to over-regularization suppressing any electrical activity during very early activation, when potentials at the body surface are at a similar level noise. As a result of the lag, a large region of tissue simultaneously activates in ECGI maps with the origin being localized to the center of this region, rather than a specific focal point as was seen in directly measured maps. It is this lag in the intrinsic deflection that is likely impacting the localization error for the ectopic origin using the AT and NPEM methods. Finding an inverse method to rectify the lag would likely improve the LE using both methods, for example, using a different regularization parameter for each moment in time rather than an average value over the QRS as was used in this study. Furthermore, as noted in the results, the CC is a poor metric in the presence of this lag and alternative metrics (such as dynamic time warping) may be preferred to evaluate the accuracy of EGM morphology.

### 4.4 Determining myocardial depth

While localizing the epicardial breakthrough of PVCs is important to guide catheter ablation procedures, it is arguably more important to determine their depth within the myocardium. This could help with pre-procedure planning by determining if epicardial access is required.

Different methods have been proposed over the years to differentiate between epicardial and endocardial origins using only epicardial surface potentials. The most well-known method hinges on the idea that recorded epicardial unipolar electrograms at the epicardial breakthrough should exhibit pure Q waves for epicardial and rS waves for endocardial sites of origin (Oster et al., 1997; Ghanem et al., 2005; Wang et al., 2011). Contrary to this, we found R-waves to be more common during epicardial pacing than endocardial pacing in directly measured data. A study using electroanatomic mapping in patients with structural heart disease (Graham et al., 2020) had similar findings to ours, being unable to detect rS complexes in the region of early activation in focal VT localized to the endocardium. Given the limitations of this method using contact mapping and the inaccuracy we found in ECGI to detect R-waves, it seems unlikely that ECGI would be able to differentiate between epicardial and endocardial origins using electrogram waveforms.

More recently, Rupp et al. (2020) demonstrated using simulated data that the size of the earliest activated region correlates to the depth of stimulus, an approach which was based on previous experimental findings by Taccardi et al. (2008). In this study, we found this method worked well in differentiating sub-epicardial and sub-endocardial paced beats for directly measured signals (see Figure 3) in particular for the LV, where the wall is thicker and even in the presence of hyperkalemia. However, ECGI did not capture this difference due to an overestimation in the size of the early activation region during sub-epicardial pacing. Despite this, the results seen with direct contact mapping are promising. The simplicity of the method makes it ideal to integrate into electroanatomic mapping systems. This could then be used to determine the depth of a focal source during endocardial mapping procedures, which could potentially help make decisions on the energy required for successful ablation or if epicardial access is needed. Further research is needed to determine specific threshold for different depths for each ventricle.

### 4.5 Limitations

The methods used to determine epicardial breakthrough sites from NPEM and AT maps were fully automated due to the volume of data. Because of this, false breakthroughs may have been detected from measured or ECGI data creating outlier LEs. However, the algorithms proved their robustness during manual verification of the automatically selected ectopic origin on NPEM and AT maps using a subset of the data for each experiment. We, therefore, do not believe these outliers have impacted the results.

To create a model of regional heterogeneity we used a local perfusion of a high K^+^ to induce hyperkalemia, a major contributor to the electrical changes seen in acute myocardial ischemia and resulting in electrically inert tissue as seen in chronic myocardial infarction. This model allowed us to compare reconstructions between healthy and pathological state in the same heart while providing a more temporally stable model than acute myocardial ischemia. However, the results here demonstrate the need to advance to using more realistic models of ischemia and infarction.

Due to the small number of R-on-T beats, statistical analysis was not possible. The examples shown do demonstrate the need to evaluate the accuracy of these beats further and the signal processing techniques that may be implemented to improve accuracy (such as subtracting the preceding T-wave). Given the similarity of reconstruction accuracy between pacing and natural PVCs, and the difficulty in obtaining data containing natural R-on-T PVCs, initial studies should concentrate on obtaining these data through pacing at various moments on the T-wave.

This study has focused on a specific inverse solution pipeline that does not necessarily represent the state-of-the art for PVC localization. There exist a multitude of different signal processing and inverse methods which are known to vary the degree of accuracy for localizing ectopic activity (Svehlikova et al., 2022). While evaluation of these different methods was not feasible in this study, the experimental data used will be made available through EDGAR (Aras et al., 2015) and can be accessed freely by other groups wishing to test their methods. In particular, it may be of interest to incorporate information about the pathological regions into the forward model such as regional reduction in conduction velocity in the zone of hyperkalemia.

Validation was performed using a torso tank experimental setup where inhomogeneous extra-cardiac electrical properties and respiration induced cardiac movement are not present. Previous studies have demonstrated the inclusion of inhomogeneous structures produces no systematic improvement in pacing site localization with ECGI (Bear et al., 2018b), but the impact on natural PVC reconstruction has never been assessed. Furthermore, it remains unclear the role of respiration-mediated cardiac movement on ECGI accuracy. Further *in vivo* validation is required.

Finally, it was not possible in this study to differentiate between PVCs originating in the myocardium or Purkinje fibers. We suspect ECGI will be less accurate in the case of PVCs originating from Purkinje fibers, as they will likely be more complex due to rapid activation of the Purkinje network. Further investigation is needed.

## Data Availability

The raw data supporting the conclusion of this article will be made available by the authors, without undue reservation.
