# Supplementary material for: Investigation into the importance of using natural PVCs and pathological models for potential-based ECGI validation
Source: Front Physiol. 2023 May 18;14:1198002. doi: 10.3389/fphys.2023.1198002 (PMC10232953; doi:10.3389/fphys.2023.1198002)
Supplement: Supplementary file 1 [file DataSheet1.PDF]

Supplementary Material

1 SUPPLEMENTARY FIGURES

1.1 Figures

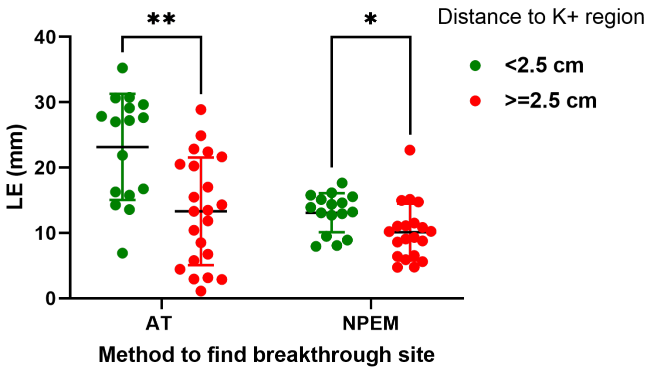

Figure S1. PVCs LE's during high K+ perfusion were separated into those close to (<25mm) and far from (>=25mm) the border of the perfusion bed for both NPEM and AT methods.
